# Arabic Gum Could Alleviate the Aflatoxin B_1_-provoked Hepatic Injury in Rat: The Involvement of Oxidative Stress, Inflammatory, and Apoptotic Pathways

**DOI:** 10.3390/toxins14090605

**Published:** 2022-09-01

**Authors:** Noha Ahmed, Samir M. El-Rayes, Waleed F. Khalil, Ahmed Abdeen, Afaf Abdelkader, Mohammed Youssef, Zainab M. Maher, Amany N. Ibrahim, Shaymaa M. Abdelrahman, Samah F. Ibrahim, Doaa Abdelrahaman, Mohammed Alsieni, Osama S. Elserafy, Heba I. Ghamry, Hanan T. Emam, Obeid Shanab

**Affiliations:** 1 Department of Chemistry, Faculty of Science, Suez Canal University, Ismailia 41522, Egypt; 2 Department of Veterinary Pharmacology, Faculty of Veterinary Medicine, Suez Canal University, Ismailia 41522, Egypt; 3 Department of Forensic Medicine and Toxicology, Faculty of Veterinary Medicine, Benha University, Toukh 13736, Egypt; 4 Center of Excellence in Screening of Environmental Contaminants (CESEC), Faculty of Veterinary Medicine, Benha University, Toukh 13736, Egypt; 5 Department of Forensic Medicine and Clinical Toxicology, Faculty of Medicine, Benha University, Benha 13518, Egypt; 6 Department of Animal Physiology, Faculty of Veterinary Medicine, South Valley University, Qena 83523, Egypt; 7 Department of Pathology and Clinical Pathology, Faculty of Veterinary Medicine, South Valley University, Qena 83523, Egypt; 8 Department of Pharmacology, Faculty of Medicine, Benha University, Benha 13518, Egypt; 9 Medical Biochemistry and Molecular Biology Department, Faculty of Medicine, Benha University, Benha 13518, Egypt; 10 Department of Clinical Sciences, College of Medicine, Princess Nourah bint Abdulrahman University, P.O. Box 84428, Riyadh 11671, Saudi Arabia; 11 Department of Pharmacology, Faculty of Medicine, King Abdulaziz University, Jeddah 22254, Saudi Arabia; 12 Department of Forensic Medicine and Clinical Toxicology, Faculty of Medicine, Cairo University, Cairo 11956, Egypt; 13 Department of Criminal Justice and Forensics, King Fahad Security College, Riyadh 13232, Saudi Arabia; 14 Department of Home Economics, College of Home Economics, King Khalid University, P.O. Box 960, Abha 61421, Saudi Arabia; 15 Department of Pharmacology, Faculty of Medicine, 6th of October University, Giza 12511, Egypt; 16 Department of Biochemistry, Faculty of Veterinary Medicine, South Valley University, Qena 83523, Egypt

**Keywords:** aflatoxin B_1_, Arabic gum, liver injury, oxidative stress, inflammatory cytokines, apoptosis

## Abstract

Aflatoxin B_1_ (AF) is an unavoidable environmental pollutant that contaminates food, feed, and grains, which seriously threatens human and animal health. Arabic gum (AG) has recently evoked much attention owing to its promising therapeutic potential. Thus, the current study was conducted to look into the possible mechanisms beyond the ameliorative activity of AG against AF-inflicted hepatic injury. Male Wistar rats were assigned into four groups: Control, AG (7.5 g/kg b.w/day, orally), AF (200 µg/kg b.w), and AG plus AF group. AF induced marked liver damage expounded by considerable changes in biochemical profile and histological architecture. The oxidative stress stimulated by AF boosted the production of plasma malondialdehyde (MDA) level along with decreases in the total antioxidant capacity (TAC) level and glutathione peroxidase (GPx) activity. Additionally, AF exposure was associated with down-regulation of the nuclear factor erythroid2–related factor2 (Nrf2) and superoxide dismutase1 (SOD1) protein expression in liver tissue. Apoptotic cascade has also been evoked following AF-exposure, as depicted in overexpression of cytochrome c (Cyto c), cleaved Caspase3 (Cl. Casp3), along with enhanced up-regulation of inflammatory mediators such as tumor necrosis factor-α (TNF-α), interleukin (IL)-6, inducible nitric oxide synthase (iNOS), and nuclear factor kappa-B transcription factor/p65 (NF-κB/p65) mRNA expression levels. Interestingly, the antioxidant and anti-inflammatory contents of AG may reverse the induced oxidative damage, inflammation, and apoptosis in AF-exposed animals.

## 1. Introduction

Aflatoxins are di-furanocoumarin derivatives generated by *Aspergillus flavus* and *A. parasiticus* strains [1]. They are unavoidable environmental pollutants due to their presence in spices, nuts, cereals, vegetables, fruits, and animal feeds, affecting both human and animal health [2]. The maximum limits of aflatoxins in foodstuffs have been regulated at 20 ppb [3]. Among the several types of aflatoxins, aflatoxin B_1_ (AF) is the most ubiquitous and the most toxic [4,5]. AF has been classified as category I carcinogen by the International Agency for Research on Cancer [6]. AF intoxication has immunosuppressive, oncogenic, teratogenic, mutagenic and genotoxic consequences on various organs [5,7]; however, liver is the principal target for AF-induced tissue injury [8]. 

AF is known to evoke tissue injury via its metabolite, namely, aflatoxin-exo-8,9-epoxide (AFO), which is an extremely reactive cytotoxic metabolite. In the hepatic microsomal system, AF undergoes an epoxidation process mediated by cytochrome-P450, where AFO is generated from AF [1,5]. AFO has a high electron affinity forming irreversible covalent bonds with nucleophilic hetero-atoms of biological macromolecules (nitrogen, oxygen, and sulfur), resulting in hepatic damage [9,10,11]. Thereafter, liver glutathione-S-transferases orchestrate the conjugation of AFO with reduced glutathione (GSH), a master endogenous antioxidant required to neutralize the AFO’s toxic potential [11,12]. Eventually, the AFO-GSH complex is subsequently metabolized in the liver to non-toxic mercapturic acid and then excreted in urine [13]. A large body of evidence has recorded the occurrence of oxidative stress after AF exposure exhibited by exhaustion of the intracellular antioxidant enzymes along with over-generation of reactive oxygen species, ROS [4,14,15]. These events trigger tissue damage confounds to AF-epoxy-DNA adduct, lipid peroxidation (LPO), protein cross-linking, and ultimately necroptosis [11,15,16]. Inflammatory pathways are reported to be a sequel to ROS overproduction [9]. Accordingly, antioxidant supplementation may be a potential therapeutic approach to withstand AF-triggered tissue damage by combating oxidative damage and promoting tissue renewal. 

Natural antioxidants have lately evoked global attention due to their substantial therapeutic efficacy and are recently widely employed as substitutional medicament [17,18]. Among these, Arabic gum (AG) is an edible gum obtained from Acacia trees which belongs to the *leguminosae* family in the tropical and subtropical regions [19,20]. AG is composed of polysaccharides, glycoproteins, and minerals such as calcium, magnesium, and potassium [21]. AG has tremendous pharmacological benefits, including antioxidant [19,22], anti-inflammatory [23], anti-obesity [24], and anti-carcinogenic [25] properties. AG antioxidant potential is attributed to its robust capability to quench the over-generated ROS, boost the oxidant scavenging system, and dampen LPO [25,26]. Furthermore, AG has an anti-inflammatory property through its ability to produce short chains of fatty acids, thus altering inflammatory cytokine production and chemotaxis in immune cells [23,27]. Accordingly, there is a plethora of literature that has elucidated the hepatic protection capability of AG toward a diversity of environmental pollutants and drugs, including CCl_4_ [28], gentamicin [29], and acetaminophen [30] intoxication.

In line with this affirmation, we anticipated that AG supplementation might mitigate AF-stimulated oxidative stress and inflammation. The current study was destined to explore the potential mechanisms of AG protection against AF-induced liver damage. Herein, a higher dose of AF than the standard regulation was used. Biochemical, hematological, and oxidative stress indices, histomorphology, and the expression of proinflammatory and apoptosis-related factors were inspected in this study.

## 2. Results

### 2.1. Biochemical Parameters Screening

**Figure 1:**
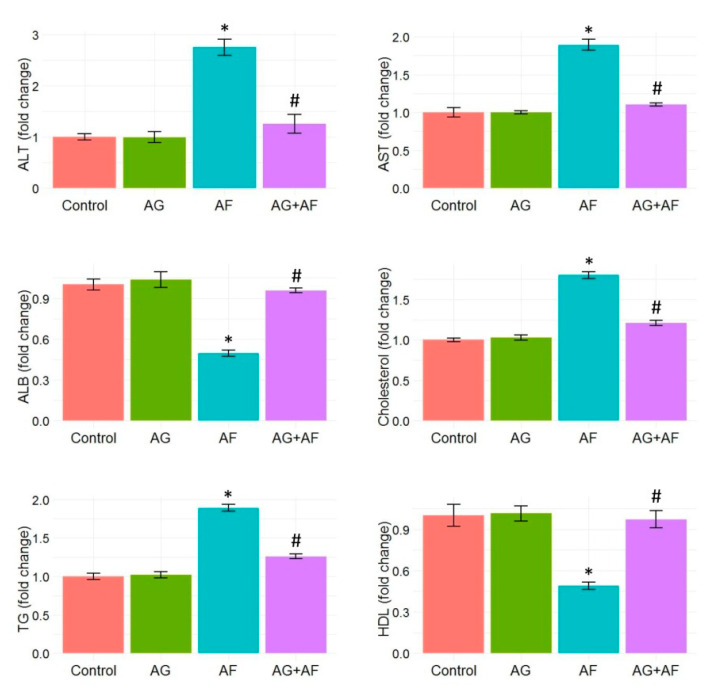
Bar plot panel of liver biochemical parameters and lipid profile upon AF and/or AG treatment. Values shown are mean ± SE (*n* = 5). ALB, albumin; ALT, alanine aminotransferase; AST, aspartate aminotransferase; HDL, high-density lipoprotein; TG, triglyceride. *p* < 0.01; * AF vs control; # AG+AF vs AF.

As depicted in Figure 1, AF exposure provoked liver injury, evidenced by a marked rise in liver transaminase activities (AST and ALT) accompanied by a noticeable reduction in the plasma albumin level compared to control rats. Additionally, we spotted a remarkable increase in TG and cholesterol plasma levels, accompanied by a dramatic reduction in HDL. These observations imply that AF exposure disrupts lipid metabolism. Conversely, preconditioning with AG robustly reduced the AF-induced injuries in liver tissues. These results are illustrated by changes in the levels of all aforementioned liver function tests and the lipid profile.

### 2.2. Hematological Profile

**Figure 2:**
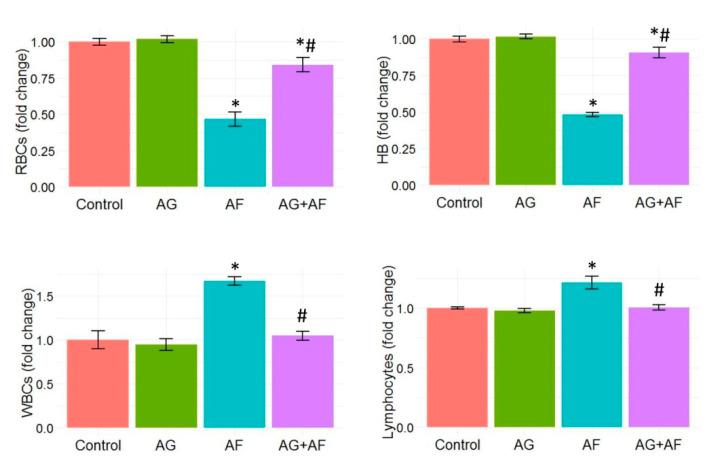
Bar plot panel of hematological profile upon AF and/or AG treatment. Values shown are mean ± SE (*n* = 5). RBC, red blood cells; HB, hemoglobin; and WBCs, white blood cells. *p* < 0.01; * AF vs control; # AG+AF vs AF.

Hb concentration and RBCs, WBCs, and lymphocyte counts following AF and/or AG treatment are displayed in Figure 2. Significant decreases in RBCs count and Hb concentration with noteworthy increases in the WBCs and lymphocyte counts following AF insult were detected. However, AG supplementation notably restored the RBCs and Hb levels in AF-intoxicated rats close to normal levels. Moreover, AG markedly reduced the elevated WBCs and lymphocyte counts in AF-treated rats.

### 2.3. Cellular Antioxidants and Lipid Peroxidation Indices

**Figure 3:**
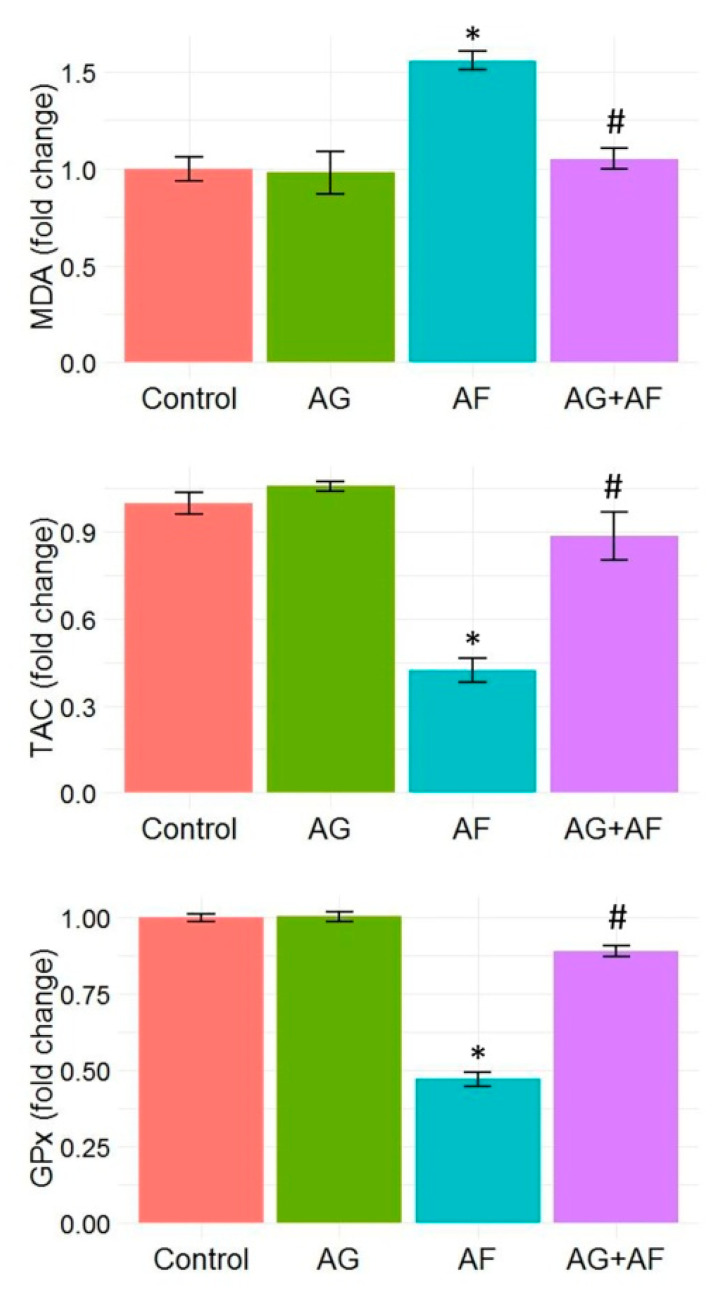
Bar plot panel of plasma antioxidant and peroxidation biomarkers changes upon AF and/or AG treatment. Values shown are mean ± SE (*n* = 5). GPx, glutathione peroxidase, MDA, malondialdehyde; TAC, total antioxidant capacity. *p* < 0.01; * AF vs control; # AG+AF vs AF.

Data of endogenous antioxidant enzymes (GPx and TAC) as well as the LPO marker (MDA) levels are shown in Figure 3. MDA levels were noticeably elevated along with substantial decreases in TAC levels and GPx activities in response to AF-intoxication. In addition, the expression levels of Nrf2 and SOD1 proteins in liver tissue showed marked down-regulation, confirming the initiation of oxidative stress (Figure 5). AF-triggered oxidative damage was considerably hampered by AG supplementation.

### 2.4. Proinflammatory Cytokine Expression

**Figure 4:**
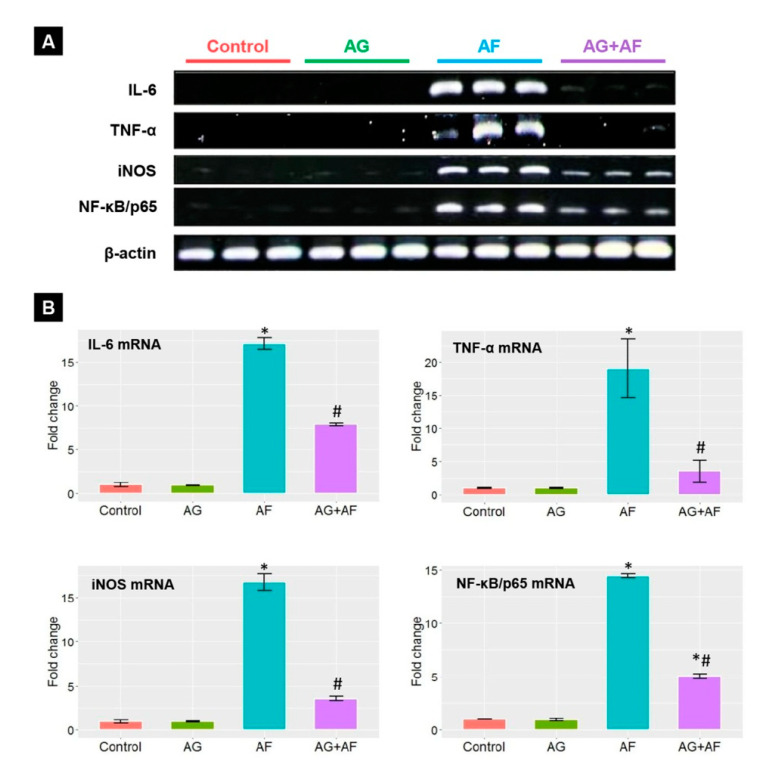
The mRNA expression of proinflammatory cytokines in liver upon AF and/or AG treatment. (**A**) representative bands for IL-6, TNF-α, iNOS, NF-κB/p65, and β-actin genes; (**B**) Bar plot panel of the semiquantitative analysis of mRNA levels of proinflammatory cytokines after normalization against β-actin. *p* < 0.01; * AF vs control; # AG+AF vs AF.

**Figure 5:**
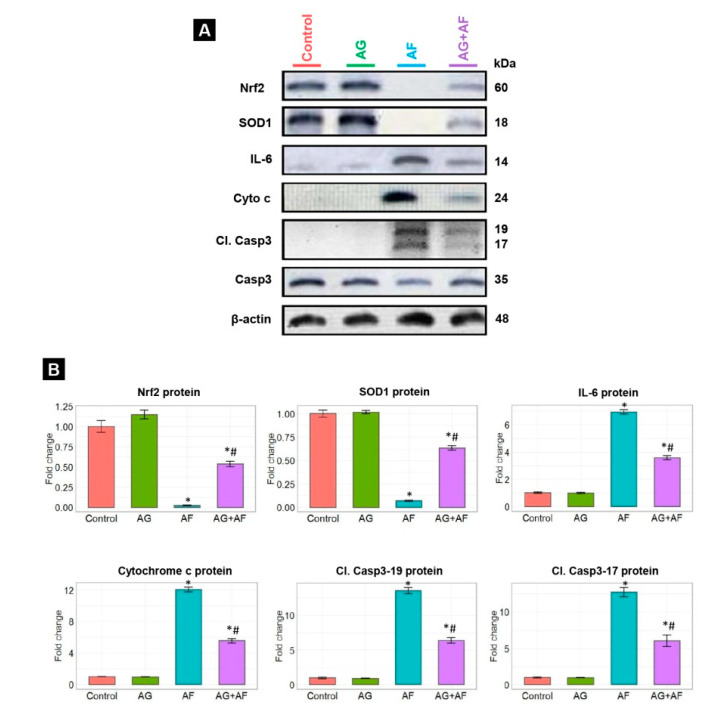
Protein expression of inflammatory cytokine, antioxidant, and apoptotic proteins in liver upon AF and/or AG treatment. (**A**) Typical immunoblots for IL-6, Nrf2, SOD1, Cyto c, Cl. Casp3-17/19, Casp3, and β-actin proteins; (**B**) Bar plot panel for semiquantitative data were created from immunoblot after normalization against β-actin. *p* < 0.01; * AF vs control; # AG+AF vs AF.

As illustrated in Figure 4, Figure 5 and Figure AF exposure stimulated the inflammatory reactions in the liver tissue seen by a drastic up-regulation of the TNF-α, IL-6, iNOS, and NF-κB/p65 mRNA expression levels along with increases in the IL-6 protein expression compared to controls. Conversely, a mitigated toxic influence of AF was observed when AF-exposed rats were treated by AG, expounded by modulation of all proinflammatory cytokines mRNA and protein expression levels. 

### 2.5. Apoptotic Biomarker Protein Expression 

Changes in protein expression of apoptotic markers in response to AF and/or AG treatments are displayed in Figure 5. AF intoxication obviously up-regulated Cyto c protein and its downstream protein, cleaved Casp3 (Casp3-17 and 19), suggesting induction of the apoptotic pathways in comparison to control rats. In contrast, we spotted controlled regulation of the expression of the same proteins in AF-insulted rats combined with AG supplementation. These results provide credence to the concept that AG was able to significantly suppress the AF-induced apoptosis in hepatic tissue.

### 2.6. Principal Component Analysis (PCA), Variable Importance in Projection (VIP) Score, and Hierarchical Clustering Heatmap

**Figure 6:**
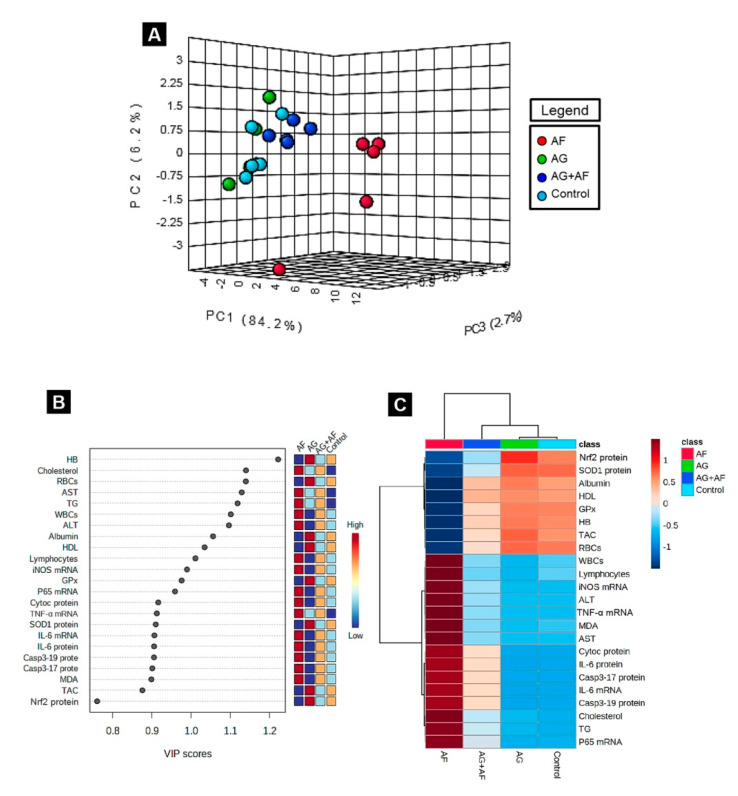
Principal components analysis (PCA) and data clustering analysis of AG versus AF-induced liver toxicity. (**A**) 3D score plot of PCA for discerning the four experimental groups (Control, AG, AF, and AG+ AF). Percentage values specified on the axes depict the contribution rate of PC1 (84.2%), PC2 (6.2%), and PC3 (2.7%) to the overall number of variations; (**B**) variable importance in projection (VIP): the colored boxes on the right display the relative concentrations of the relevant measured parameters in each study group, while, the contribution intensity is indicated by a colored scale spanning from the highest (red) to lowest (blue); (**C**) hierarchical clustering heatmap enables intuitive visualization of all data sets. Each colored cell on the map denotes the concentration values, with variable averages in rows and different treatment sets in columns. Dark red is the highest value on the gradation scale, and blue represents the lowest value.

Next, to uncover the relationships between different treatments and variables, PCA was conducted. The PCA showed that all variables were incorporated into three main principal dimensional components (PC1, PC2, and PC3), accounting for 93.1% of the total variance. Most of the examined variables were distinguished by PC1, and hence described the greater proportion of variance (84,2%), whereas the lower proportion of variance was reflected by PC2 (6.2%) and PC3 (2.7%). The PCA revealed that the Control, AG, and AG+AF groups were clustered together on the left side of the gel and segregated from those treated with AF (mainly along the PC1 dimension; Figure 6A). Moreover, the variable importance in projection (VIP) indicated that cholesterol, TG, AST, ALT, WBCs, lymphocytes, MDA, iNOS mRNA, NF-κB/p65 mRNA, TNF-α mRNA, IL-6 mRNA, Casp3 protein, Cyto c protein, and IL-6 protein were the most influential variables in the discrimination of AF-treated animals from the rest (Figure 6B). The clustering heatmap illustrated in Figure 6C provides an intuitive visualization of all the data sets, which summarizes the marked difference between the concentration values of all measured parameters in response to AF toxicity compared to the other treated groups. These findings demonstrate that the AF-exposed animals showed more damage than animals in the other groups.

### 2.7. Hepatic Histoarchitecture Inspection

**Figure 7:**
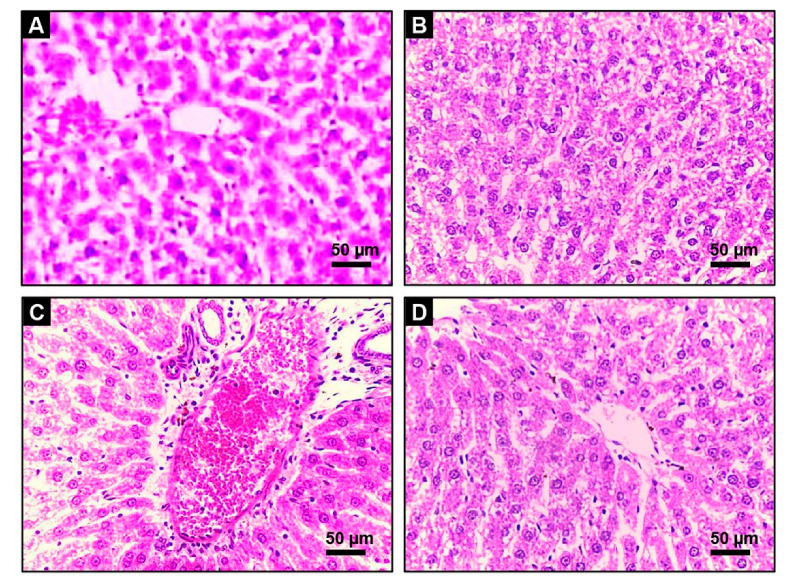
Histopathology of liver tissue in Control, AG, AF, and AG+ AF-treated groups. Grossly normal architecture of hepatic lobules was observed in the Control (**A**), and AG-treated (**B**) rats; AF (**C**) liver section of AF-treated group showed periportal cytoplasmic vacuolation with fatty degeneration, severe hemorrhage, bile duct hyperplasia, and inflammatory cellular infiltration; AG+ AF-treated group (**D**) exhibited substantial improvement in hepatic architecture, indicated by mild fatty vacuolation and portal inflammation.

To emphasize the previously noted findings, the histological modulation in the hepatic tissue upon AF and/or AG administration was investigated. The hepatic histological examination of the Control and AG-treated rats exhibit normal construction of liver lobule (homogenous polyhedral liver cells, well-organized sinusoids, and portal veins) presented in Figure 7A,B, respectively. In contrast, the AF-treated rats exhibited a centrilobular vacuolation of hepatocyte cytoplasm with fatty degeneration. Additionally, severe central venous congestion associated with bile ducts and Kupffer cells hyperplasia were also detected. Patches of necrosis with significant inflammatory cellular leakage in the liver portal regions were also spotted (Figure 7C). Alternatively, after the administration of AF with AG, the portal zone displayed minimal inflammatory cells spillage with barely noticeable centrilobular changes. The hepatic architecture mostly returned to normal with very minor fatty aberrations (Figure 7D).

## 3. Discussion

AF is one of the most harmful toxins that commonly contaminate grains, spices, food, and feedstuffs and remains stable throughout food processing, thus posing substantial health concerns [7,31]. Compelling evidence strongly suggests that LPO, excessive oxidant generation, cellular antioxidant inadequacy, and mitochondrial alteration are deemed po be fundamental mechanisms implicated in their pathogenesis [4,9,32]. 

According to previous reports, the liver is the central organ affected during aflatoxicosis, where AF is chiefly bio-transformed into a potent secondary toxic intermediate (AFO) [1,5]. AFO has the aptitude to promptly attach to the cellular biomolecules such as RNA, DNA, as well as other protein constituents, provoking tremendous generation of ROS such as superoxide anions (O_2_^•–^), hydroxyl radicals (OH^•^), hydrogen peroxide (H_2_O_2_), and nitric oxide (NO) intracellularly [10]. Notably, GSH is the key sovereign antioxidant that abundantly exists in all biological systems and is essential for quenching the AFO damaging effect via the formation of AFO-GSH conjugates as well as scavenging the generated ROS [11]. Consequently, the GSH store is depleted, causing perturbation of cellular redox homeostasis and acceleration of LPO [4,11,15]. Herein, the current results emphasized that oxidative stress, and increased LPO played a crucial role in AF-induced hepatic injury. This was evident in decline in the activities of GPx and TAC content alongside marked elevations of MDA levels in rats exposed to AF. Additionally, the Nrf2 and SOD1 protein expressions were downregulated. It is well known that Nrf2 signaling pathway is a key modulator of cellular detoxification process and the redox state [33], which helps boost cellular antioxidant capacity shown by the upregulation of SOD1 expression [7]. Interestingly, GPx is an endogenous antioxidant enzyme that serves as the initial line of enzymatic antioxidant defense essential for catalyzing H_2_O_2_ into O_2_ and H_2_O and reduction of LPO [34,35,36]. Thus, the observed increase of the LPO marker (MDA) in the present data may be attributed to the AF-induced reduction in the GPx activity and exhaustion of the antioxidant defense mechanisms [36]. Even worse, MDA itself has the ability to significantly alter the mitochondrial membrane potential, cellular proteins, and DNA integrity resulting in extensive cellular damage [4]. As expected, the elevated LPO compromised the membrane integrity of the liver cells, increasing their permeability, enabling hepatic enzymes (transaminases) to be released into the circulation and consequently elevating their plasma levels, thus uncovering the existence of hepatic dysfunction [10]. In accordance with our data, Hua et al also reported a significant impairment of hepatic function with increased liver enzyme activity following AF exposure [37]. Our histopathological examination of liver sections vividly mirrored this biochemical finding. 

Furthermore, we observed disruption of lipid metabolism, as revealed by increases of cholesterol, TG, and HDL levels providing evidence of substantial hepatocellular deterioration in response to the induced aflatoxicosis [32]. These findings are in agreement with those reported by Aleissa et al. [10] who also observed a significant increase in cholesterol level after AF exposure in a rat model. In addition, parallel to the previous research, our study revealed decreased albumin concentration in the AF-treated animals [14,38]. The reduction in albumin confirmed the occurrence of liver dysfunction which may have been influenced by ROS-induced DNA and protein degradation that in turn inhibited the transcription and translation processes [37]. 

Moreover, our findings demonstrated hepatocellular damage represented by a substantial decrease in hemoglobin and RBCs. During liver damage, protein synthesis is inhibited, lowering the albumin levels, iron-binding capacity, hematopoietic factors (iron, folic acid, and vitamin B12), and the synthesis of erythropoietin. Consequently, the erythrocyte synthesis was inhibited [6,39,40]. Another potential contributory factor for anemia is AF-stimulated erythrocyte oxidative stress, which changes membrane permeability and induces erythrocyte hemolysis [6].

According to accumulating data, inflammation is strongly associated with oxidative distress [41,42]. Therefore, we hypothesized that the inflammatory pathway is another possible mechanism involved in AF-induced hepatic injury [43]. Oxidative stress and increased ROS generation are thought to enable the proinflammatory gene expression and release of the inflammatory cytokines stimulating the inflammatory response [16]. In the current investigation, AF significantly enhanced the TNF-α, IL-6, iNOS, and NF-κB/p65 expression in liver tissue which may contribute to the induction of inflammation. Notably, upregulation of mRNA expression of these inflammation-related genes, has been formerly observed after AF exposure in various studies [15,33,35,44]. TNF-α is the earliest and most important inflammatory mediator involved in the pathogenesis of inflammation [45,46]. TNF-α and NF-κB/p65 pathways trigger the activation of proinflammatory cytokines, including IL-6 and iNOS, together with adhesion molecules which stimulate the recruitment of leukocytes at the site of inflammation [39,42,47,48,49]. 

Unfortunately, iNOS promotes the production of NO as well as the formation of toxic peroxy-nitrite species (ONOO^−^) which have an affinity for cellular biomolecules and adversely enhance inflammatory reaction and cell death [50,51]. These data were confirmed by our histopathological examination, where marked inflammatory cell infiltrations in hepatic tissue were demonstrated along with significant increases in total WBCs and lymphocytic counts.

Increasing evidence suggests that AF stimulates the apoptotic signaling pathway by induction of mitochondrial and oxidative stress and inflammation [7,15,35,43]. It has been documented that AF induces irreversible mitochondrial membrane injury leading to the liberation of Cyto c into the cytosol; thereby, the Casp3 is activated with its downstream apoptotic proteins initiating the apoptotic cascade [52,53]. In accordance, the current investigation revealed a dramatic up-regulation of apoptotic biomarkers (cleaved Casp3-17, Casp3-19, and Cyto c) in the liver tissue [54]. Therefore, together with previous studies, our data strongly suggest the involvement of apoptotic pathways in AF-induced hepatic damage [7,55].

AG, is an edible proteinaceous polysaccharide plant product widely used in Arab folk medicine for its renowned antioxidant, anti-inflammatory, and immunomodulatory capabilities [56]. The antioxidant property is owing to its content of amino acid residues such as lysine, tyrosine, and histidine as well as branched chains of polysaccharides [22,24]. There is copious evidence corroborating the idea that AG consumption increases the activity of antioxidant enzymes through modulation of the expression of oxidative stress genes, including Nrf2 [19,22], along with mitigation of LPO [56,57]. In addition, AG has been reported to exhibit potent anti-inflammatory effects via suppuration of TNF-α, iNOS expression, monocyte chemotactic protein-1, and IL-6 [19,23,37]. Moreover, AG is a rich source of bioavailable short-chain fatty acids produced in substantial quantities by intestinal microbiota’s fermentation of AG, mainly butyrate, that play a crucial function in suppressing the expression of proinflammatory cytokines [26,56,57,58]. The current histopathological examination revealed a decreased inflammatory cell infiltration in liver tissues when AG was provided to AF-intoxicated animals. 

In addition, The current findings agree with a growing body of literature that supplementation with AG lowers plasma total cholesterol and TG concentrations by increasing the intestinal content viscosity which, in turn, reduces intestinal absorption of lipids. An additional mechanism proposes that soluble fibers enhance the secretion of bile acids and thus decrease plasma cholesterol levels [24]. Furthermore, AG supplementation also showed an anti-apoptotic effect against AF-induced up-regulation of Casp3 expression [59].

**Figure 8:**
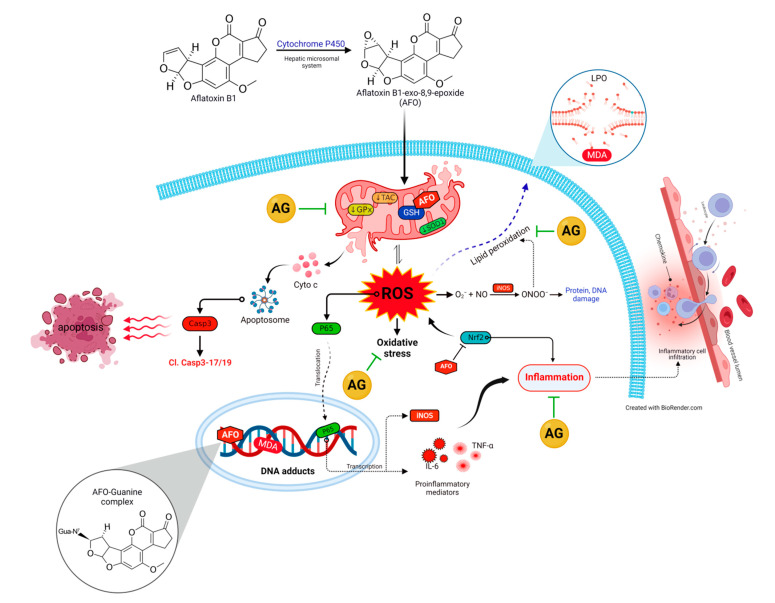
The molecular insights behind the protective effect of AG following AF-induced toxicity. AFO, aflatoxin-exo-8,9-epoxide; AG, Arabic gum; Cl. Casp3-17/19, cleaved Caspase3-17/19; Cyto c, cytochrome c; GPx, glutathione peroxidase; GSH, reduced-glutathione; IL-6, interleukin-6; iNOS, inducible nitric oxide synthase; LPO, lipid peroxidation; MDA, malondialdehyde; NF-κB/p65, nuclear factor kappa-B transcription factor/p65; NO, nitric oxide; Nrf2, nuclear factor erythroid2–related factor2; ONOO^−^, toxic peroxy-nitrite species; ROS, reactive oxygen species; SOD1, superoxide dismutase1; TAC, total antioxidant capacity; TNF-α, tumor necrosis factor-α.

To summarize the various contributions through different interventions on the liver tissue, we adopted multivariate statistical analysis represented by PCA. Each treatment was mainly discriminated along the PC1 axis (84.2%). AF-treated animals could be markedly differentiated from the other groups because they were clustered on the right side of the gel, away from other treatments. However, the AG+AF co-exposed group was clustered close to the Control and AG groups. The clustering heatmap illuminated noticeable differences between the concentration values of all variables in response to AF exposure compared to the other treatment groups. Hence, a higher dose of AF than the standard international regulation was used in this experiment. These data strongly confirm the potential protective effect of AG against AF intoxication. The molecular insights underlying the protective effect of AG following AF-induced toxicity are outlined in Figure 8.

## 4. Conclusions

AF can provoke notable liver damage caused by oxidative stress, lipid peroxidation, and inflammatory reactions. AG supplementation has the capability to abrogate the hepatic cells from AF-induced damage. These improvements are suggested to be attributed to AG’s antioxidant, anti-inflammatory, and anti-apoptotic properties. Our data advocate food supplementation with AG owing to both preventive and remedial activities against AF-triggered liver damage.

## 5. Materials and Methods

### 5.1. Animals and Protocol of Trial

Male Wister albino rats weighing 120 ± 10 g, aged 9 w old, were used for performing this trial. Rats were purchased from the Center of Laboratory Animals, Faculty of Veterinary Medicine, Assiut University, Egypt. Rats were accommodated in convenient normal conditions for a two weeks prior to the experiment (temperature ∼25 °C). Throughout the experiment, all animals were fed a standard basal diet as well as given water ad libitum. The Ethics Committee of the Faculty of Veterinary Medicine, South Valley University approved the use of experimental animals and the study design (Approval no. 31).

Subsequent to acclimatization, experimental animals were designated to 4 equivalent groups (5 rats each). Control group; AG group, rats were given AG dissolved in the drinking water at dose of 7.5 g/kg b.w (2 mL for each rat, orally once a day) [60]; AF group which set as a positive toxic group, where, animals were exposed to AF (dissolved in saline) at dose rate 200 µg/kg b.w, orally day after day [61], representing about 6.7 ppm in feedstuffs; and AG+AF group, in which rats were given the forementioned doses of AG and AF. Noteworthy, AG was administrated 5 h before AF exposure. The experiment was continued for 28 sequential days. AF (purity ≥ 99%) and AG were purchased from Sigma Aldrich (St Louis, MO, USA).

### 5.2. Specimens Collection and Processing

On the 28th day of the trial, a 3–4 mL blood sample was retrieved from the retro-orbital venous plexus and centrifuged at 3000× *g* for 15 min; the plasma was collected and then maintained at −20 °C for biochemical bioassay. Next, all animals were sacrificed under the conventional protocol of inhalation anesthesia (isoflurane), and the liver was rapidly dissected and washed out with ice-cold physiological saline to scrub away clotted blood, and cut into several portions. One portion was fixed in 10% formalin for further histopathological analysis. Other fresh tissue parts were employed for RNA/protein extraction and kept within −80 °C. The remaining portions of fresh tissue were preserved at −20 °C for sequent evaluation of oxidative cascade marker.

### 5.3. Biochemical Parameters Bioassay and Hematological Profile

The gathered plasma was employed for assessment of the liver function biomarkers, including aspartate aminotransferase (AST), alanine aminotransferase (ALT), and albumin (ALB). Moreover, triglycerides (TG), total cholesterol, and high-density lipid (HDL) were assessed. All procedures were accomplished in accordance with the manufacturer’s (Laboratory Biodiagnostics Co., Giza, Egypt) recommendations.

Hemoglobin concentration (Hb), RBCs, WBCs, and lymphocyte counts were determined for the whole blood samples using an automated blood analyzer (Urit-2900 plus, Urit Medical Electronic Co., Shenzhen, China).

### 5.4. Antioxidants and Peroxidation Biomarkers

The plasma levels of oxidative biomarkers were evaluated, including malondialdehyde (MDA), total antioxidant capacity (TAC) and glutathione peroxidase (GPx). All parameters were according to the manufacturer’s protocol (Laboratory Biodiagnostics Co., Cairo, Egypt).

### 5.5. Reverse Transcription-PCR

Total RNA was isolated from liver homogenate employing QIAzol Lysis Reagent (QIAzol™, QIAGEN^®^, MD, USA) following the manufacturer’s instructions. The quantity and quality of total RNA in the samples were checked with a spectrophotometer (NanoDrop ND-1000 Spectrophotometer, Thermo Scientific, MA, USA). The RNA quality was estimated by the 260/280 nm absorbance ratio. The isolated total RNA was reverse transcribed into cDNA using miScript II RT kit (QIAGEN^®^, MD, USA). The cDNA was synthesized from 1 μg RNA using a random primer (oligo(dT) primers, PrimeScript™, TaKaRa Bio Inc, CA, USA). A thermal cycler (A200 Gradient Thermal cycler, LongGene^®^, Hangzhou, China) was used to perform the PCR employing the listed primers in Table 1. The PCR was achieved after 30 cycles of 95 °C/30 s, 60 °C/30 s, and 72 °C/1 min. Next, the yielded PCR products were electro-phoretically segregated in an ethidium bromide-stained 1.5% agarose gel (Scientific Limited, Northampton, UK) in tris-borate-EDTA (TBE) buffer. All separated bands were observed by a gel recording system (Bio-Rad, CA, USA) and band strength was quantified and standardized to β-actin using the NIH Image J software (version 1.47, NIH, MD, USA).

### 5.6. Western Blotting

Protein fraction extracted from the organic phase of QIAzol Reagent-processed fatty tissue samples (QIAGEN^®^, QIAzol™) following the manufacturer’s guidelines, treated with a proteinase inhibitor cocktail (Sigma-Aldrich, Steinheim, Germany) and phosphatase inhibitor tablet (PhosStop™, Roche Diagnostics, IN, USA). Protein specimens were loaded in equal amounts, separated using SDS-poly acrylamide gel (SDS-PAGE) electrophoresis, and then blotted on a polyvinylidene difluoride membrane (Immobilon™-P PVDF membrane, Merck Millipore, MA, USA).The membranes were blocked in PBS–Tween (0.1%) with 1% BSA and were then probed with the diluted primary antibodies (as listed in Table 2). The Lumi-light Plus kit from Roche and the BioRAD chemidoc were used to detect the bands. Intensities of bands were assessed with the NIH Image J software. 

### 5.7. Histoarchitectures Assessment

The harvested liver tissue sample was fixed in 10% formalin for 24 h. Next, the specimen was washed under running tap water for ten minutes and soaked in sequent dilutions of ethanol (70, 80, 95, and 100%) to dehydrate. Following dehydration and before being embedded in paraffin, samples were cleared in xylene. The prepared blocks were sliced into 4 μm thick sections using a microtome. Then, all sections were stained with hematoxylin and eosin (H&E) for screening and images, using a camera integrated digital imaging system (DM300, Leica, Wetzlar, Germany).

### 5.8. Statistical Data Analyses

Data analyses were implemented using one-way analysis of variance (ANOVA) followed by Duncan’s post hoc test employing SPSS software (Version 21; SPSS Inc., Chicago, IL, United States) for comparison of treatment means. All values are expounded as the mean and 95% confidence interval and considered statistically relevant at *p* ≤ 0.05. The “ggplot2” package was installed in in RStudio (R version 4.0.2) to implement the bar plots. Moreover, a 3Dplot for the multivariate principal component analysis (PCA), variable importance in projection (VIP) score, clustering heatmap were generated using the MetaboAnalyst software. 

## Figures and Tables

**Table 1:** Forward and reverse primers sequences.

Primers	Forward (from 5′ to 3′)	Reverse (from 3′ to 5′)	Accession No.
NF-κB/p65	GACGAGGCTCGGAGAGCCCA	CTGGGGCGGCTGACCGAATG	NM_001029913.1
iNOS	CACCACCCTCCTTGTTCAAC	CAATCCACAACTCGCTCCAA	NM_012611.3
IL-6	TCCTACCCCAACTTCCAATGCTC	TTGGATGGTCTTGGTCCTTAGCC	NM_012589.2
TNF-α	AAATGGGCTCCCTCTCATCAGTTC	TCTGCTTGGTGGTTTGCTACGAC	X66539.1
β-actin	AGGCACCAGGGTGTGAT	ATGTCACGCACGATTTCC	NM_031144.3

**Table 2:** Listing of used antibodies.

Antibody	Manufacturer	Catalog No.	Clone No.	Dilution Factor
Nrf2	SANTA CRUZ	Sc-518036	H-10	1/1000
SOD1	SANTA CRUZ	Sc-101523	24	1/1000
Cytochrome c	Cell signaling	12963S	6H2.B4	1/1000
IL-6	Cell signaling	12912	D5W4V	1/1000
Caspase3	Cell signaling	#9662		1/1000
Cleaved-Caspase3 (Asp175)	Cell signaling	#9664	5A1E	1/1000
β-actin	Sigma-Aldrich	A5441	AC-15	1/1000
Goat anti-rat IgG/HPR	Abcam	ab205720		1/1000

## Data Availability

Upon request, the data utilized to verify the findings of this research are obtainable from the corresponding authors.

## Abbreviations

AF, aflatoxin B_1_; AFO, aflatoxin-exo-8,9-epoxide; AG, Arabic gum; ALB, albumin; ALT, alanine aminotransferase; AST, aspartate aminotransferase; Cl casp3-17/19, cleaved caspase3-17/19; Cyto c, cytochrome c; HDL, high-density lipid; GPx, glutathione peroxidase; GSH, reduced glutathione; IL-6, interleukin-6; iNOS, inducible nitric oxide synthase; LPO, lipid peroxidation; MDA, malondialdehyde; NF-κB/p65, nuclear factor kappa-B transcription factor/p65; NO, nitric oxide; Nrf2, nuclear factor erythroid2–related factor2; ONOO−, toxic peroxy-nitrite species; ROS, reactive oxygen species; SOD1, superoxide dismutase 1; TAC, total antioxidant capacity; TG, triglycerides; TNF-α, tumor necrosis factor-α.
